# Chlorine-Doped Graphitic Carbon Nitride for Enhanced Photocatalytic Degradation of Reactive Black 5: Mechanistic and DFT Insights into Water Remediation

**DOI:** 10.1021/acsomega.5c04017

**Published:** 2025-08-27

**Authors:** Jau-Min Ji, Tesfaye Abebe Geleta, Yang-hsin Shih, Ren Qian Tee

**Affiliations:** Department of Agricultural Chemistry, 33561 National Taiwan University, No. 1, Sec. 4, Roosevelt Road, Taipei 106, Taiwan

## Abstract

Photocatalysts are recognized as eco-friendly technologies that exhibit significant potential for removing organic pollutants upon exposure to light. Herein, we successfully modified graphitic carbon nitride (CNM) by chlorine (Cl) doping through a calcination process to enhance the photocatalytic degradation of Reactive Black 5 (RB5) under visible-light irradiation. The Cl-doping efficiency was comprehensively assessed, with CNM-Cl(0.4) demonstrating the best photocatalytic performance, achieving a rate constant of 0.199 min^–^^1^, which is 1.76 times higher than that of undoped CNM. The observed enhancement can be ascribed to the improved photocurrent response and the narrowing of the bandgap, both of which result from the incorporation of chlorine into the CNM framework. The incorporation of Cl into CNM resulted in more than double the photocurrent generation compared to bare CNM, promoting rapid charge carrier separation and significantly reducing charge recombination. This was further supported by BET surface area analysis, where Cl doping led to a ∼4-fold increase in specific surface area, facilitating more active sites for pollutant adsorption. Additional information about the electronic characteristics of CNM and CNM-Cl was obtained through first-principles density functional theory (DFT) calculations, which confirmed the experimental findings. The photocatalytic degradation mechanism is carried out by the production of reactive oxygen species, such as hydroxyl radicals (•OH) and superoxide anions (•O_2_^–^). The results of this study show that chlorine doping greatly improves the photocatalytic performance of carbon nitride materials (CNM). This modification makes CNM a very promising metal-free photocatalyst for environmental remediation and water purification under visible light irradiation, especially considering its high stability, reusability, and eco-friendly synthetic approach.

## Graphical abstract

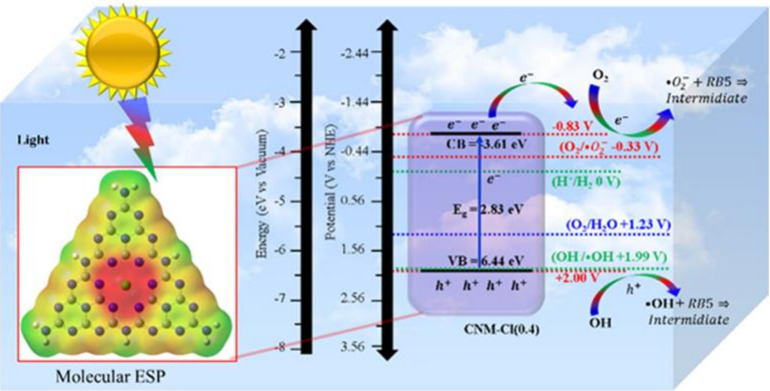

## 1 Introduction

The disposal of organic dyes poses significant risks to human health, aquatic ecosystems, water quality, and soil fertility, often leading to reduced seed germination. According to a 1990 Environmental Protection Agency (EPA) report, chemical runoff from agricultural soils accounts for 50% of river and wastewater pollution. These pollutants not only harm the environment, but also increase the risk of human toxicity.^1,2^ Therefore, the elimination of these contaminants from wastewater is critical and can be effectively addressed through various approaches, including membrane filtration, adsorption techniques, advanced oxidation processes, biological treatments, and photocatalytic degradation, each of which plays a pivotal role in advancing nanomaterial-based catalytic technologies.^3–5^ The choice of materials or catalysts used in wastewater treatment significantly affects the effectiveness of these processes, making the development of efficient materials a central research focus.

In recent years, two-dimensional (2D) materials have attracted considerable research interest, with particular focus on graphitic carbon nitride (g-C_3_N_4_)—a polymeric semiconductor primarily composed of carbon and nitrogen atoms.^6,7^ Typically synthesized by introducing nitrogen into carbon-rich precursors, g-C_3_N_4_ has emerged as a promising material due to its broad applicability across various fields. Notably, it has demonstrated promise as a metal-free photocatalyst owing to its suitable band gap (approximately 2.7 eV, which enables activity under visible light irradiation.^8^ These characteristics make them suitable for various applications including chemical sensors,^9,10^ energy harvesting,^11^ hydrogen evolution,^12^ water splitting,^13^ biomedical applications,^14^ and pollutant degradation.^15^ Moreover, g-C_3_N_4_ exhibits robust thermal stability, good electrical conductivity, chemical stability, and enhanced charge carrier mobility.^16–20^ Nevertheless, the photocatalytic performance of g-C_3_N_4_ under visible-light illumination is often constrained by its inherently limited light-harvesting capability and relatively low specific surface area, which collectively hinder its overall efficiency.^21,22^ Studies have consistently highlighted the critical role of g-C_3_N_4_ nanostructure morphology in the photocatalytic performance. Moreover, studies have highlighted that both the selection of precursor materials for g-C_3_N_4_ synthesis and the concentration of dopants play crucial roles in determining its crystallographic properties and photocatalytic performance.

However, the effective use of electron–hole pairs under illumination has been significantly limited by a number of inherent restrictions, such as a relatively low specific surface area, inadequate absorption of visible light, and fast recombination of photogenerated charge carriers.^23^ Researchers have used a variety of techniques targeted at enhancing charge carrier dynamics and speeding up reaction kinetics in order to get over these obstacles and improve photocatalytic activity. These strategies include surface morphology engineering,^24^ heterojunction system construction,^25^ and elemental doping.^26^ Among these strategies, elemental doping is an effective method for tuning the inherent energy-band structure. Recent reports have highlighted significant progress in modifying g-C_3_N_4_ to enhance its photocatalytic performance, particularly through supramolecular self-assembly and defect/dopant engineering elemental doping and morphology control.^27^ Element doping typically involves the introduction of metal elements such as Fe,^28^ Li,^29^ K,^30^ Na,^31,32^ and Ni^33^ as well as nonmetal elements including S,^34^ P,^35^ O,^36^ and Cl.^37^

Although several studies have reported on Cl-doped g-C_3_N_4_,^37,38^ critical aspects such as the optimization of the Cl-to-melamine ratio for achieving maximum photocatalytic efficiency, the elucidation of the degradation mechanism, and theoretical validation through computational modeling remain insufficiently addressed. Therefore, the present study aims to synthesize Cl-doped g-C_3_N_4_ via a controlled calcination method and systematically investigate its photocatalytic behavior through a combination of experimental techniques and density functional theory (DFT) calculations. This integrated approach is intended to provide a comprehensive understanding of the structural and electronic modifications induced by Cl incorporation and their implications for photocatalytic performance under visible light irradiation.

## 2 Experimental Section

### 2.1 Materials

Melamine (C_3_H_6_N_6_, 99%) was acquired from Alfa Aesar, and ammonium chloride (NH_4_Cl, 99.5%) was procured from Riedel-de Haën. Sodium chloride (NaCl, ≥99.5%) and sodium hydroxide (NaOH, 98.7%) were sourced from J.T. Baker (USA). Methanol, ethanol, and hydrochloric acid (HCl, 37%, reagent-grade) were obtained from Scharlau. Sigma-Aldrich (USA) provided sulfuric acid (H_2_SO4) and Reactive Black 5 (RB5). All aqueous solutions were made using ultrapure water with a resistivity of 18.2 MΩ·cm, which was obtained from a Milli-Q water purification system (Millipore, Bedford, MA, USA). All experimental procedures were carried out under ambient conditions.

### 2.2 Synthesis of g-CN

Melamine serves as a distinct precursor for synthesizing g-C_3_N_4_ through direct pyrolysis in a muffle furnace.^13,39,40^ Typically, melamine is calcined at a high temperature, specifically at 550 °C, and is referred to as CNM throughout this manuscript. Because of the stability of the melamine structure, when subjected to high-temperature calcination in a muffle furnace, approximately 80% of the CNM product was obtained.

### 2.3 Synthesis of Cl-doped g-CN

Cl-doped CNM were prepared via a high-temperature calcination process, as described in ref 41. In details, melamine (1.0 g) was meticulously combined with different quantities of ammonium chloride (0.1–0.5 g) using a mortar to achieve uniformity. After that, the mixture was heated to 550 °C at a rate of 1 °C per minute in a covered crucible. For 4 h, the temperature remained steady. The yellow solid that formed when the mixture was allowed to naturally cool to room temperature was then crushed into a fine powder and utilized in further testing. The sample obtained with 0.1 g of NH_4_Cl was denoted as CNM–Cl(0.1), as shown in Figure S1. Similarly, additional samples were synthesized with increasing NH_4_Cl contents of 0.2, 0.3, 0.4, and 0.5 g, and were labeled as CNM–Cl(0.2), CNM–Cl(0.3), CNM–Cl(0.4), and CNM–Cl(0.5), respectively.

### 2.4 Preparation of RB5 Pollutant

An appropriate concentration of RB5 was dissolved in deionized water and sequentially diluted to generate a calibration curve (Figure S2). A calibration curve was used to determine the actual concentration during the successive degradation of RB5 after visible light irradiation using the prepared catalyst. The absorbance of the RB5 dye was measured at a wavelength of 595 nm using UV–vis spectroscopy to establish the detection point and evaluate its photocatalytic degradation performance.

### 2.6 Adsorption Test

Adsorption of RB5 onto the prepared catalysts was conducted in the dark. The prepared catalysts (250 mg/L) were mixed with 50 ppm RB5 in an aqueous solution and subjected to ultrasonic vibration for 1 min. The mixture was kept in the dark with constant stirring for 30 min. At regular intervals, the samples were filtered using a syringe filter, and adsorption was determined by measuring the UV–vis absorbance. Once the adsorption of RB5 onto the catalyst surface reached equilibrium, its photocatalytic capability was evaluated under visible-light exposure. To evaluate RB5 removal, a control experiment was conducted using RB5 alone (without catalyst). The amount of RB5 adsorbed onto the prepared catalysts was determined by comparing the initial and final concentrations of RB5 in the solution.

### 2.7 Characterization of Catalysts

A comprehensive set of analytical techniques was employed to characterize the synthesized catalysts. These included scanning electron microscopy (SEM), X-ray diffraction (XRD), and X-ray photoelectron spectroscopy (XPS, ESCAPHI1600 model). The specific surface areas of the catalysts were measured using nitrogen adsorption with an Accelerated Surface Area and Porosimetry System (ASAP 2010, Micromeritics) and calculated using the Brunauer–Emmett–Teller (BET) method. Additional characterization involved ultraviolet–visible spectroscopy (UV–vis, CT-2200 spectrophotometer), diffuse reflectance UV–vis spectroscopy (UV–vis DRS, JASCO V-670), and dynamic light scattering (DLS), electron paramagnetic resonance (EPR) and electrochemical impedance spectroscopy (EIS, CHI614D electrochemical workstation). An ultrasonic cleaner (Delta DC400) was used during sample preparation.

### 2.8 Computational Method

First-principles density functional theory (DFT) calculations were conducted utilizing the hybrid B3LYP functional,^42,43^ which is widely acknowledged for its reliability in predicting molecular geometries and electronic properties, as well as its consistency with experimental observations in analogous systems. Because of the balance between computational efficiency and accuracy, B3LYP was deemed suitable for the objectives of this study. Geometry optimizations were conducted using the 6-311G basis set^44^ in the Gaussian 09 software suite. The resulting optimized molecular structures were visualized using GaussView 5.0.8.^45^ The optimized geometries were subsequently utilized to examine the frontier molecular orbitals, with a particular focus on the highest occupied molecular orbital (HOMO) and the lowest unoccupied molecular orbital (LUMO). Additionally, the molecular electrostatic potential (ESP) was visualized on the molecular surface at an isosurface value of 0.02 to examine the charge distribution. The GaussSum3.0 software package was employed to generate the density of state (DOS) spectra for the CNM and CNM-Cl structures, providing insights into the occupied and virtual orbitals.^46^

## 3 Results and Discussion

### 3.1 Characterization of the Photocatalysts

SEM analysis was used to investigate the surface morphology of the synthesized catalysts. Figure 1A shows the morphologies of the CNM and CNM–Cl. In Figure 1A­(a), the CNM shows a planar layered structure with noticeable material aggregation between layers, with particle sizes ranging from 2 to 8 μm. In Figure 1A­(b–e), the SEM morphology of CNM-Cl reveals reduced aggregation compared to that of the undoped CNM nanosheet. Notably, the planar layered structure exhibited a slight curvature at the edges, with an average particle size ranging between 4 and 6 μm. The addition of Cl to the CNM resulted in the formation of smaller and more evenly distributed particles. This is due to the decomposition of NH_4_Cl during the high-temperature calcination process, which produces gases such as ammonia (NH_3_) and hydrogen chloride (HCl). These gases may contribute to the peeling off of the bulk CNM, leading to smaller grain sizes with a noticeable increase in Cl concentration, as shown in Figure 1A­(b–e). This is expected to increase the number of active sites available for the reaction.^47,48^

**Figure 1:**
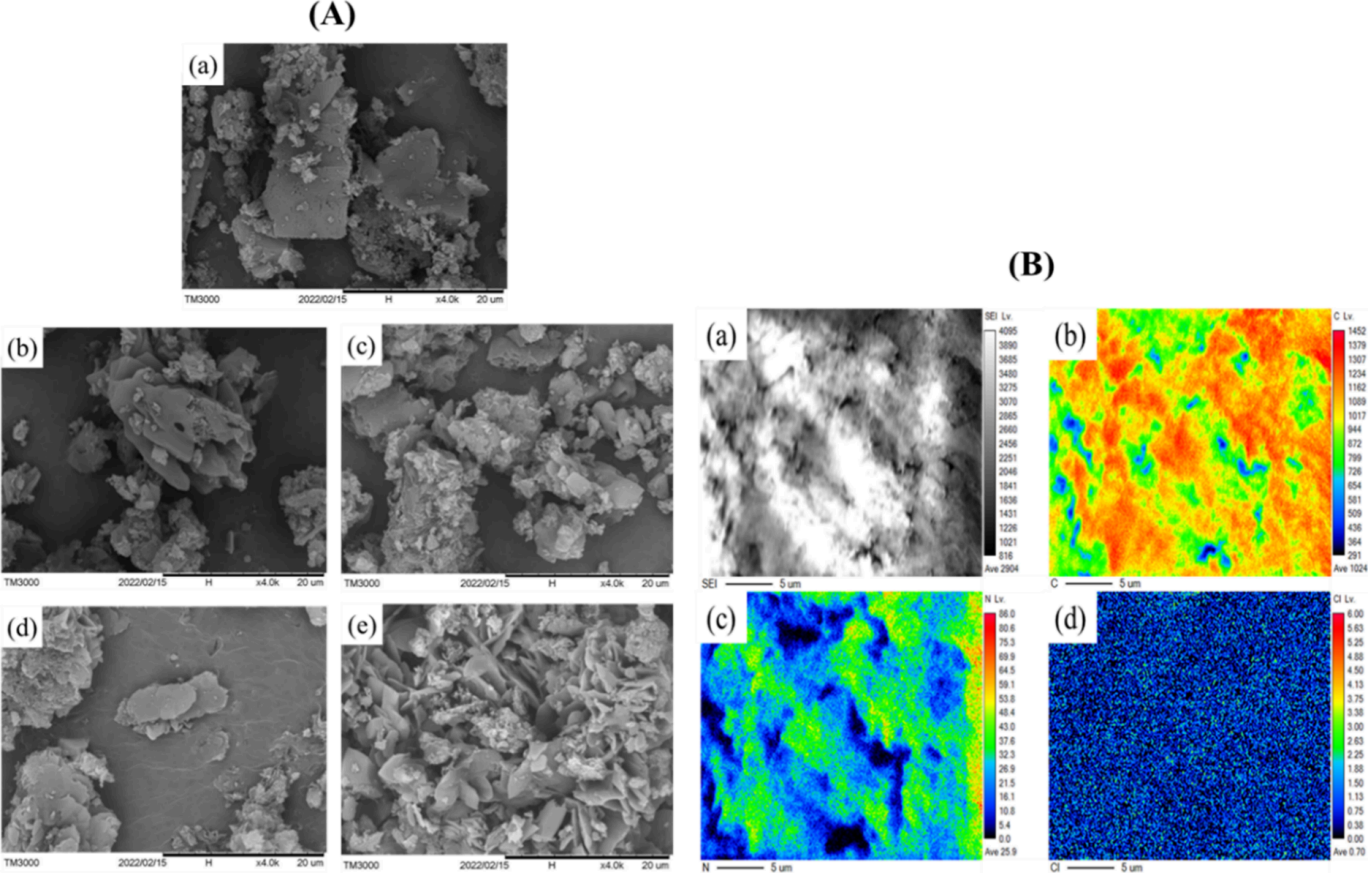
(A) SEM images of (a) CNM, (b) CNM–Cl(0.2), (c) CNM–Cl(0.3), (d) CNM–Cl(0.4), and (e) CNM–Cl(0.5) photocatalysts. (B) Electron probe microanalyzer (EPMA) mapping analysis of (a) CNM–Cl(0.4), scanning electron microscopy of (b) C, (c) N, and (d) Cl atoms.

Elemental mapping of CNM–Cl(0.4) was conducted using an Electron Probe Microanalyzer (EPMA) to assess the spatial distribution and composition of elements within the catalyst. Figure 1B­(a) shows the scanned image of the CNM–Cl(0.4) used for the subsequent elemental analysis. The analysis showed that the catalyst consisted mainly of C and N atoms with a minor presence of Cl atoms, as shown in Figure 1B­(b,c,d). Although the chlorine concentration was very low, its distribution in the material was uniform (Figure 1B­(d)).

XRD analysis was conducted to examine the crystalline structure of both pristine and chlorine-doped CNM. The XRD patterns of the prepared g-C_3_N_4_ exhibit two characteristic peaks at 13.4° and 27.9° (assigned to the (100) and (002) planes), consistent with the standard graphite-like structure (JCPDS No. 87-1526) as shown in Figure 2a and in agreement with previous literature.^49,50^ The diffraction peak at 13.4°, corresponding to the (100) plane, is relatively weak and is attributed to the in-plane periodic arrangement of tri-s-triazine units. The reduced intensity is indicative of surface defects and increased disorder, resulting in a more amorphous and less planar morphology. In contrast, the strong (002) peak at 27.9° is a hallmark of graphitic-like layered structures, reflecting π–π stacking between conjugated aromatic layers.^51^ As the Cl concentration in the CNM increased, a reduction in the peak intensity was observed. This suggests that the inclusion of Cl may have caused a reduction in the grain size of the as-prepared CNM.^52^ From the XRD analysis, we concluded that Cl doping occurred interstitially as no peak shift was observed. Nevertheless, a higher Cl doping concentration results in a gradual reduction in the diffraction peak intensity.^48^

**Figure 2:**
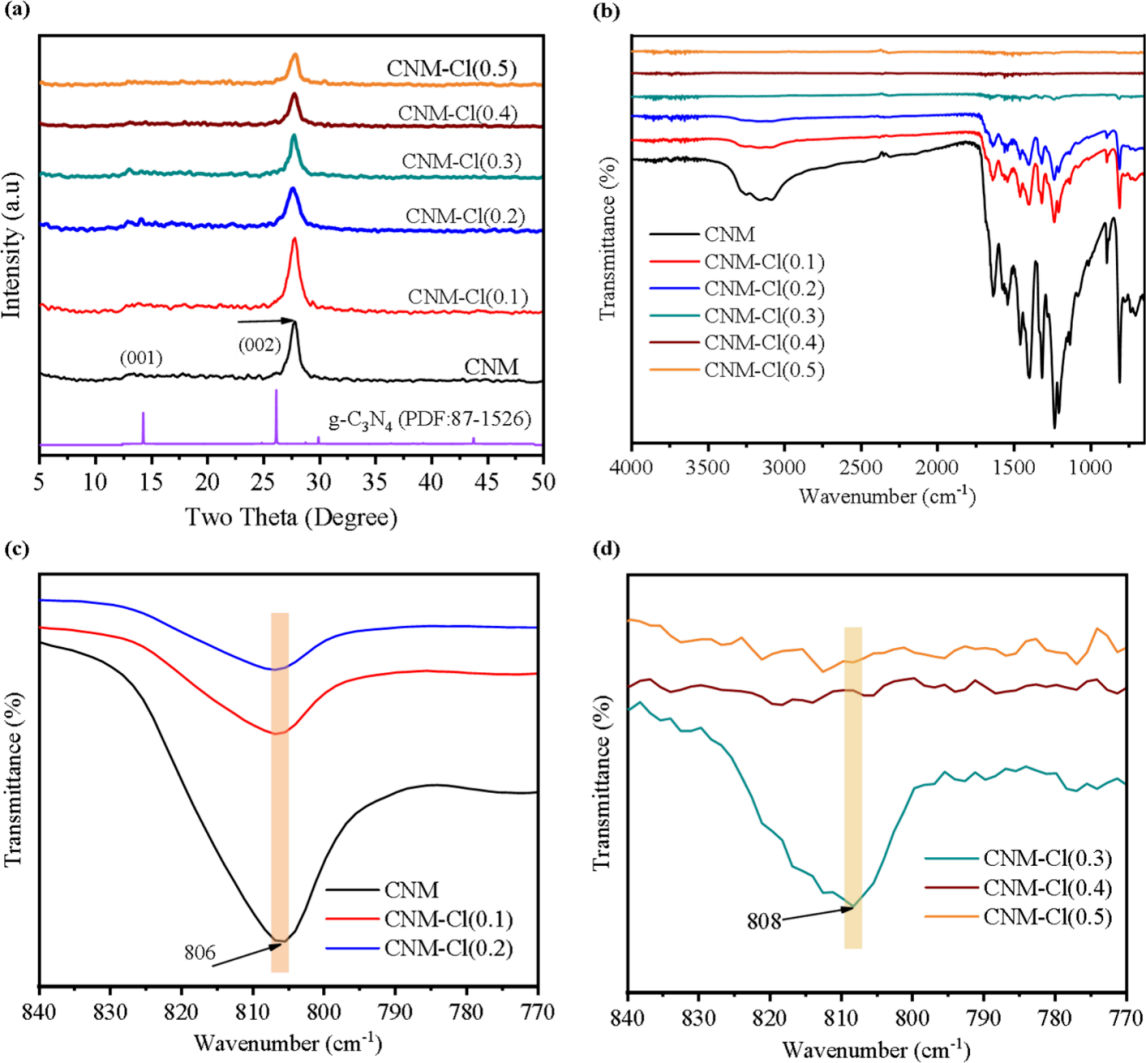
(a) X-ray diffraction (XRD) patterns and (b–d) Fourier-transform infrared (FTIR) spectra of Cl-doped CNM­(CNM–Cl).

FT-IR spectroscopy was utilized to examine the functional groups in the synthesized materials. Figure 2b illustrates the FT-IR spectra for both CNM and CNM–Cl.FT-IR spectroscopy was employed to investigate the functional groups present in the synthesized materials. As shown in Figure 2b, the FT-IR spectra of both CNM and CNM–Cl are displayed. A broad transmission band observed within the range of 3000–3500 cm^–^^1^ is attributed to the N–H stretching vibrations and hydroxyl (O–H) groups, indicating the presence of surface-adsorbed water molecules.^53,54^ Moreover, multiple peaks ranging from 1150 to 1750 cm^–1^ were identified as characteristic C–N heterocyclic stretches,^55^ indicating the presence of a C–N network in the samples. Additionally, as depicted in Figure 2c, the peak at 806 cm^–1^ confirms the presence of a tri-s-triazine structure in the synthesized catalysts.^56^ Notably, the observations in Figures 2c,d reveal that, as the Cl concentration increased, the peak associated with the tri-s-triazine structure diminished. This reduction in crystallinity suggests that excess Cl may affect the grain size of CNM, which is consistent with the XRD data.

The CNM–Cl(0.4) catalyst’s surface elemental composition was investigated using XPS. The survey spectrum confirms that carbon (C), nitrogen (N), and chlorine (Cl) are present in the sample matrix, as shown in Figure 3a. A tiny oxygen (O) signal additionally came up, which was probably caused by ambient oxygen being adsorbed during the heat polymerization process. Two separate peaks at binding energies of 284.69 and 288.10 eV can be seen in the high-resolution C 1s spectra of CNM–Cl(0.4), which is displayed in Figure 3b. These peaks are attributed to aromatic carbon species (C–C/C=C) and sp^2^-hybridized carbon atoms within the heptazine units (N–C=N), respectively.^57,58^ Additional peaks at 293.73 and 286.07 eV correspond to C–N and C–O bonds, respectively, with the latter likely resulting from surface oxidation due to air exposure. The N 1s spectrum reveals four distinct peaks associated with different nitrogen environments: pyridinic N (398.54 eV), pyrrolic N (399.65 eV), graphitic N (400.87 eV), and nitrogen oxide species at 404.38 eV.^59–61^ In the case of the CNM–Cl(0.4) catalyst, pyridinic N was observed at a higher relative content than the other peaks of the nitrogen species (Figure 3c). Pyridinic N, with its lone pair of electrons, serves as an effective coordination site for the formation of N–Cl bonds. The Cl 2p XPS spectrum depicted in Figure 3d confirms the presence of Cl ions in CNM–Cl(0.4) at 198.94 eV, indicating Cl doping in the carbon nitride material.^62^ These XPS results suggest that Cl is present in the CNM structure as a noncovalently bonded species. The Cl 2p binding energy (∼198.94 eV) aligns with interstitial Cl^–^ ions rather than covalent Cl–C or Cl–N bonds, which typically appear at higher binding energies. Furthermore, the absence of new peaks or significant shifts in FTIR spectra and the unaltered peak positions in XRD patterns further support that Cl is not chemically bonded but rather intercalated or located within pores. These findings are consistent with previously reported Cl-doped g-C_3_N_4_ systems where chloride is incorporated interstitially without forming direct bonds with the host lattice.^63^

**Figure 3:**
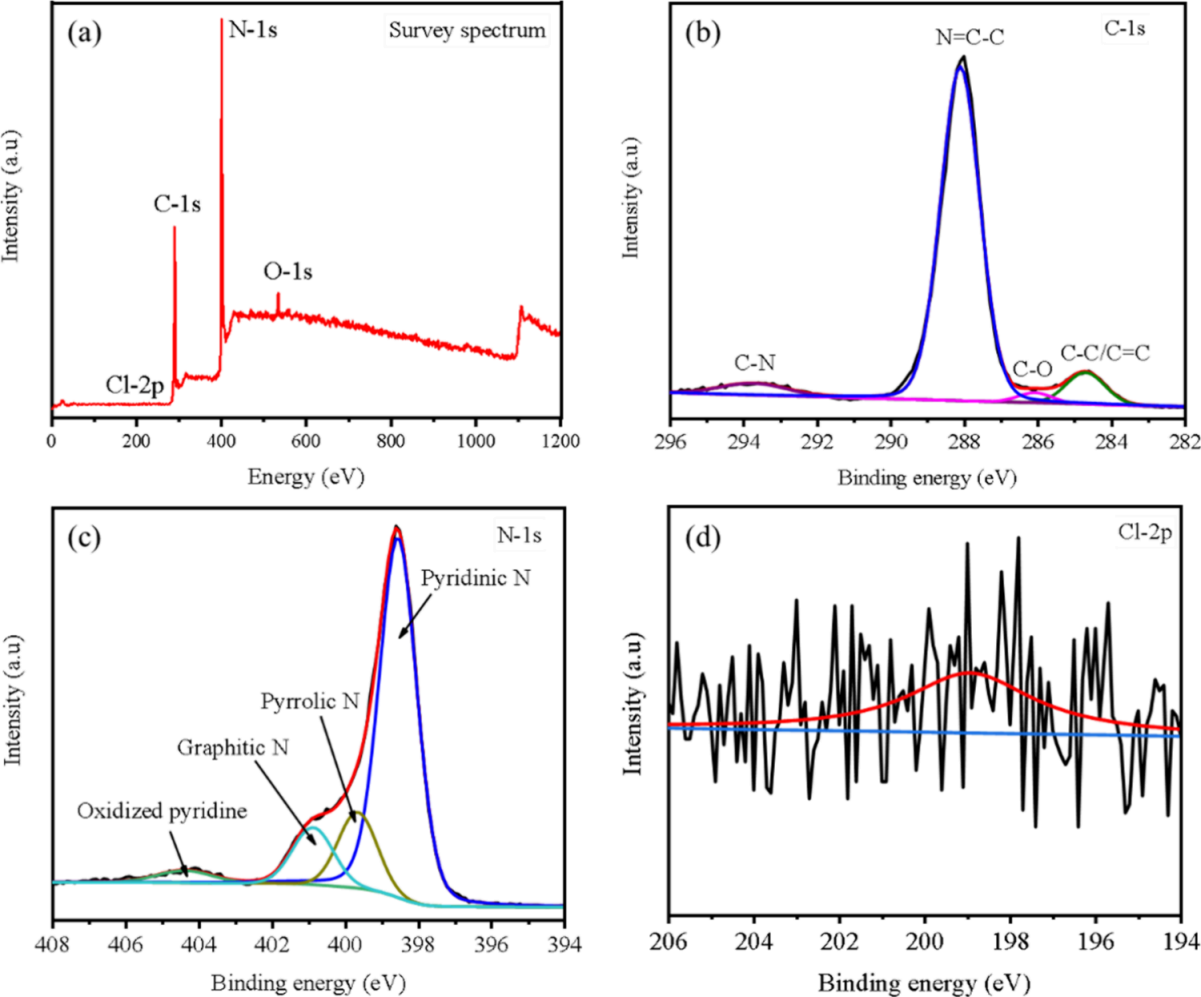
(a) XPS survey spectrum of CNM–Cl(0.4), along with high-resolution spectra of (b) C 1s, (c) N 1s, and (d) Cl 2p regions for the same sample.

To evaluate the influence of Cl-doping on the surface area of the photocatalysts, the specific surface areas were determined using nitrogen adsorption–desorption isotherms and analyzed via the BET method. The undoped CNM showed a surface area of 11.22 m^2^/g, whereas CNM-Cl(0.4) exhibited a significantly higher surface area of 44.68 m^2^/g (Table S1). This notable increase is attributed to the exfoliation effect and porosity introduced during NH_4_Cl decomposition, which leads to smaller particle size and enhanced surface exposure. The improved surface area of CNM-Cl(0.4) facilitates greater active site exposure and efficient adsorption of RB5 molecules, contributing to its superior photocatalytic activity.

DLS analysis (Figure 4a) revealed the particle size distribution of Cl-doped CNM dispersed in aqueous solution. The pristine CNM exhibited an average particle size of approximately 1100 nm, while Cl incorporation led to a notable reduction in particle size, decreasing to approximately 600 nm. The synthesized materials’ optical behavior was assessed using UV–vis DRS. The addition of chlorine caused a little redshift of the absorption edge, as seen in Figure 4b. This is probably related to changes in the electronic band structure. Notably, the CNM–Cl(0.4) sample exhibited stronger absorption within the visible spectrum, suggesting superior capability for capturing visible light.

**Figure 4:**
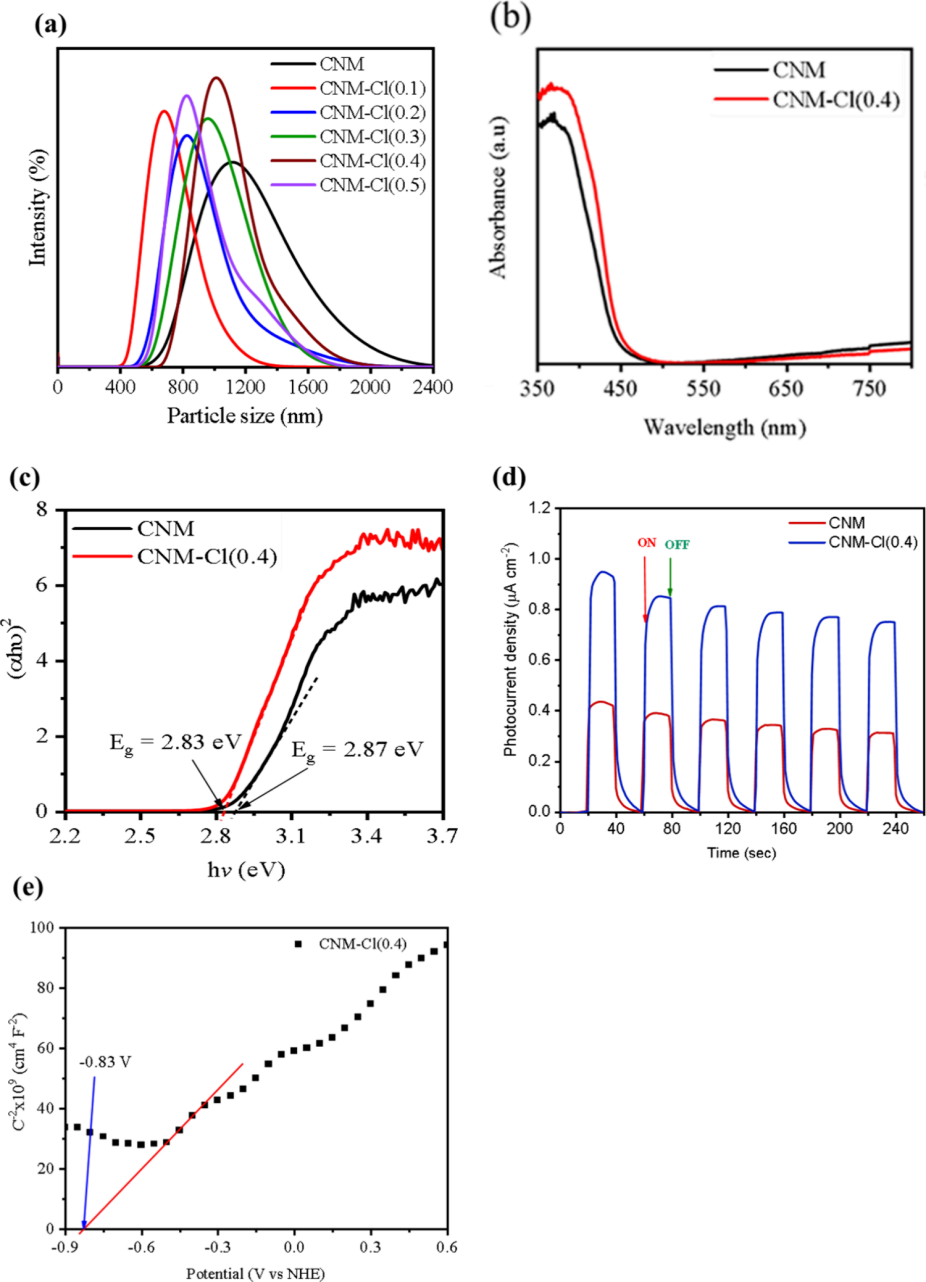
(a) DLS particle size distribution of CNM–Cl nanosheet photocatalysts, (b) UV–Vis diffuse reflectance spectroscopy (DRS), and (c) Tauc plot for CNM and CNM–Cl(0.4) catalyst. (d) Transient photocurrent responses excited by the visible light irradiation of CNM and CNM-Cl(0.4); and (e) Mott–Schottky plot of CNM-Cl(0.4).

Based on the DRS absorption spectra, the band gaps of bare CNM and Cl-doped CNM were determined using the Tauc plot equation.^64–66^ The resulting Tauc plot, shown in Figure 6c, indicates that the bandgap of CNM (2.87 eV) decreases slightly when Cl atoms are incorporated, with CNM–Cl(0.4) exhibiting a bandgap of 2.83 eV (Table S1). The results of related investigations are in line with this band gap decrease brought about by the addition of halogen atoms, especially chlorine.^67,68^ The composite catalyst’s photocatalytic activity is improved and charge carrier separation is made easier by the smaller bandgap.

### 3.2 Electrochemical Measurement

The kinetics of photoinduced charge transfer were investigated in a 0.1 M Na_2_SO_4_ electrolyte solution with an applied bias potential of 0.45 V vs Ag/AgCl. As illustrated in Figure 4d, the photocurrent measurements provide valuable insights into the generation and transport behavior of photogenerated charge carriers. Transient photocurrent responses were recorded for both pristine CNM and Cl-doped CNM (CNM–Cl(0.4)) by periodically illuminating the samples with intermittent light exposure, alternating between light ON and OFF states in 20 s intervals over a total duration of 260 s. The CNM–Cl(0.4) sample demonstrated a markedly higher photocurrent density than the undoped CNM, suggesting improved photoresponse, enhanced photon absorption capability, and more effective separation and mobility of charge carriers.^69,70^

Figure 4e illustrates the Mott–Schottky analysis of CNM–Cl(0.4), revealing a positive slope that confirms its n-type semiconducting properties. The flat-band potential for CNM–Cl(0.4) was approximately −0.827 V when measured against a normal hydrogen electrode (NHE). Through UV–vis DRS, the optical bandgap energy (*E*_g_) was determined to be 2.83 eV. By integrating this bandgap value with the flat-band potential, the conduction band (CB) and valence band (VB) positions were calculated to be −3.61 and −6.44 eV, respectively, relative to the vacuum level.

Additionally, by correlating data from UV–vis DRS and Mott–Schottky measurements, the VB potential was estimated to be 2.00 V versus NHE. These electronic properties suggest that CNM–Cl(0.4) exhibits appropriate redox potentials for driving the photogeneration of reactive oxygen species, including the reduction of O_2_ to •O_2_^–^ and the oxidation of OH^–^ to •OH, as conceptually represented in Scheme 1.

**Scheme 1:**
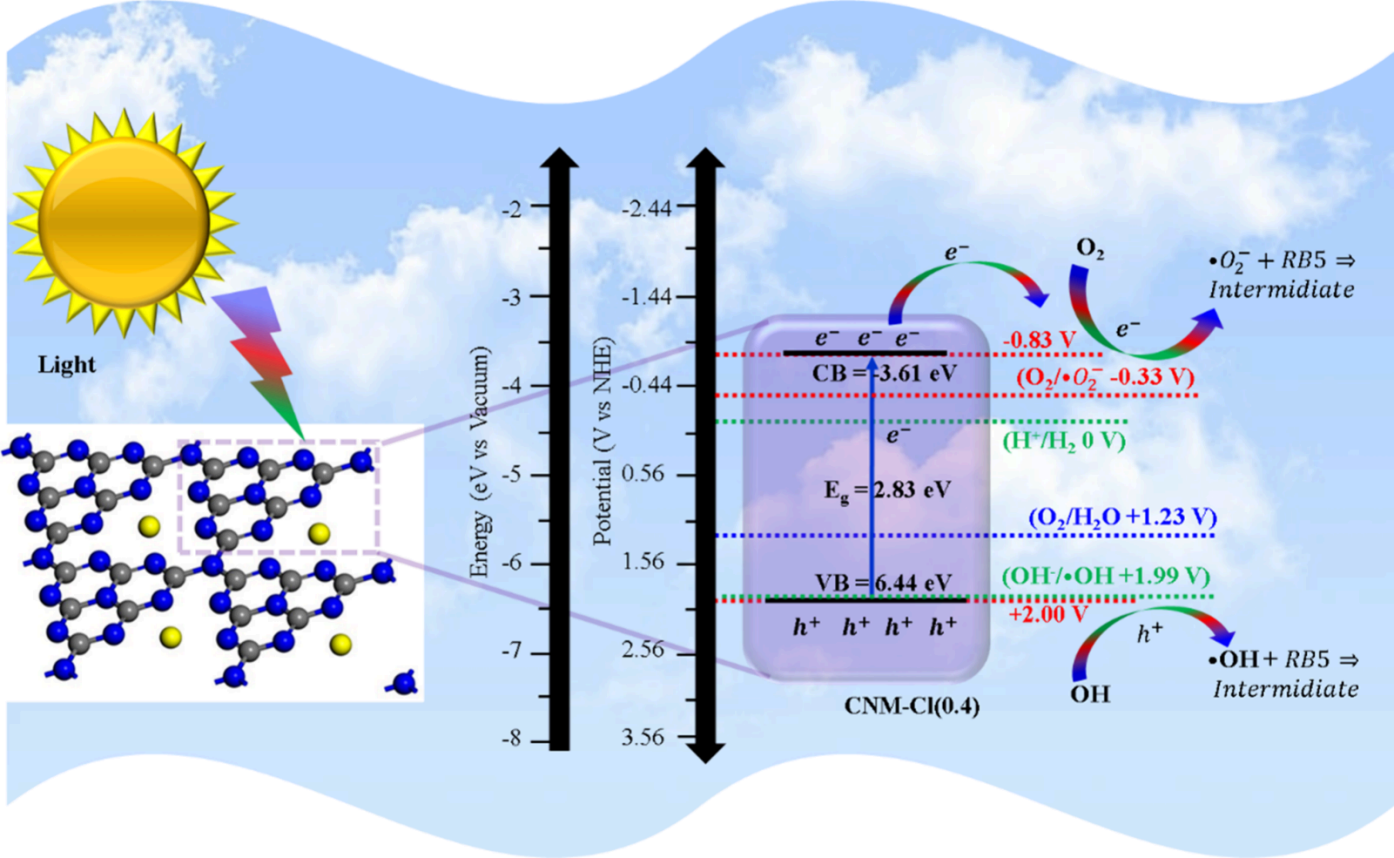
Illustration of the Charge Carrier Dynamics, Depicting the Separation of Photogenerated Electron–Hole Pairs in the CNM-Cl(0.4) Photocatalyst under Visible Light Exposure

### 3.3 Photocatalytic Performance on the Degradation of RB5 Pollutant

Prior to assessing the catalyst’s performance, it is advised that the pollutant’s stability be assessed under visible light exposure. In our work, pollutant RB5 shown significant stability during visible-light exposure in the absence of a catalyst. This stability served as the control curve for pollutant degradation assessments after catalyst injection, as shown in Figure 5a. The photocatalytic efficiency of the Cl-doped CNM nanosheets for RB5 degradation is illustrated in Figure 5a. The figure shows that when CNM was used alone, RB5 was completely degraded in approximately 40 min. However, the incorporation of Cl–atoms into the bulk CNM nanosheet significantly accelerated RB5 degradation, reaching 96, 99, and 99% degradation within 25 min for CNM–Cl(0.3), CNM–Cl(0.4), and CNM–Cl(0.5), respectively. This improvement was attributed to the smaller particle size resulting from the high-temperature treatment of CNM and NH_4_Cl, as revealed by the SEM micrographs and DLS spectra. These analyses indicate a decrease in grain size and a narrower particle size distribution, leading to an increase in active sites, better charge separation, and lower recombination rates.^71,72^

**Figure 5:**
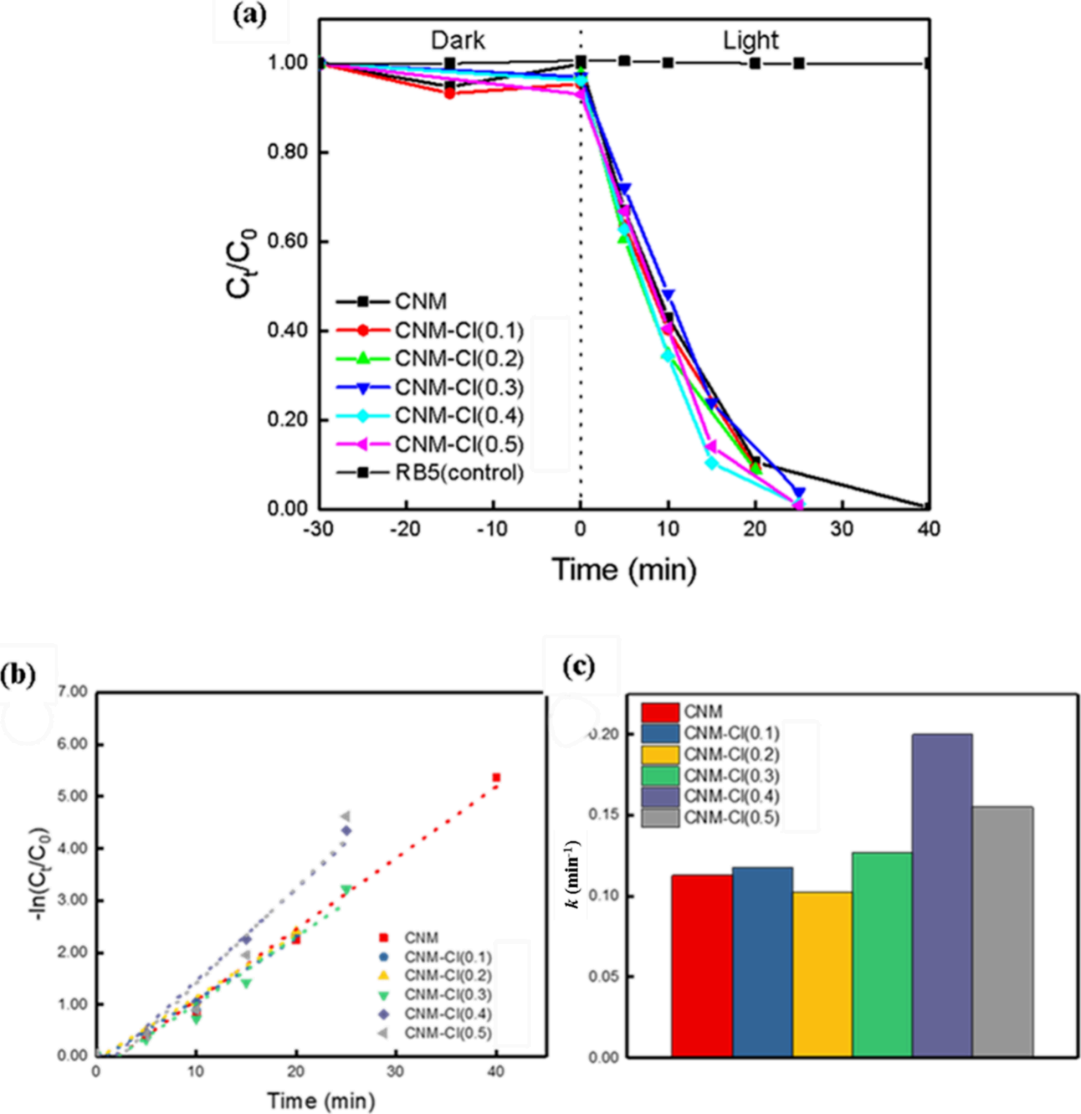
(a) Photocatalytic degradation, (b) pseudo-first-order kinetic reaction, and (c) the corresponding rate constants of Cl-doping CNM for the removal of RB5 pollutant. ([Catalyst]: 250 mg/L; [RB5]: 50 ppm; light source: 420 nm LED, 11300 Lux).

To further investigate the efficiency of Cl–doping in the CNM catalyst, we applied the pseudo-first-order model and illustrated the corresponding rate constants in Figure 5b,c. Notably, CNM–Cl(0.4) exhibited a rate constant (*k*) value of 0.199 min^–1^, exceeding that of CNM (0.113 min^–1^) by 1.76 times (Table S1), leading to efficient charge carrier separation and enhanced photogeneration occurs in the presence of Cl-atoms in the CNM. Therefore, the CNM–Cl(0.4) catalyst was identified as the most effective for this investigation.

To further confirm the stability and reusability of the CNM-Cl(0.4) photocatalyst, a recycling test was performed across three successive cycles of RB5 degradation under identical conditions (Figure S3). CNM-Cl(0.4) maintained nearly 100% degradation efficiency in the first and second cycles and approximately 70% in the third cycle. In contrast, the undoped CNM catalyst showed a notable decline in performance, with only ∼33% degradation in the third cycle. These results demonstrate the superior stability and reusability of the CNM-Cl(0.4) catalyst, corroborating its potential for practical wastewater treatment. Similar enhancements in catalyst longevity have been reported in related works.^73,74^

Table S2 provides a comparative summary of various photocatalysts used for the degradation of RB5 under different experimental conditions. Among the catalysts listed, CNM-Cl(0.4), developed in this study, demonstrates the highest photocatalytic performance. It achieves a degradation efficiency of 99% within just 25 min, with a remarkably high rate constant of 0.199 min^–^^1^. This is significantly superior to other reported catalysts, such as rGO (0.01395 min^–^^1^, 56% degradation in 60 min), TiO_2_-coated PET (0.0328 min^–^^1^, 99.99% in 120 min), WO_3_/TiO_2_ (0.0439 min^–^^1^, 92% in 120 min), and Ag/ZnO (0.0017 min^–^^1^, 74% in 780 min). Notably, CNM-Cl(0.4) maintains excellent activity even at a higher RB5 concentration (50 ppm), compared to lower concentrations used in other studies (10–30 ppm), while utilizing a moderate catalyst dosage of 250 mg/L. The enhanced performance under visible-light irradiation (150 W LED) highlights the effectiveness of CNM-Cl(0.4) as a metal-free, sustainable photocatalyst for environmental remediation. In contrast, other catalysts either require longer reaction times, higher dosages, or less practical light sources such as UV-A. These findings indicate that CNM-Cl(0.4) not only exhibits faster reaction kinetics but also holds great promise for real-world applications in wastewater treatment.

### 3.4 DFT Calculation of Electronic Properties

To further validate the experimental results, we calculated the electrochromic properties using DFT. A finite cluster model of g-C_3_N_4_, consisting of three heptazine units with hydrogen-terminated edges, was used in the DFT calculations. This nonperiodic approach, commonly applied in Gaussian-based studies,^75,76^ enables efficient analysis of localized electronic effects. While it does not capture full structural periodicity, it adequately reflects the electronic modifications induced by Cl doping, consistent with experimental results. The optimized structures of the CNM and CNM-Cl are shown in Figure 6a,d, respectively. The experimental XRD patterns clearly demonstrate that incorporating Cl atoms into the CNM reduces the peak intensity without causing a peak shift, indicating that Cl doping occurs at interstitial sites. Therefore, we placed Cl atoms in interstitial positions within the tri-s-triazine structure for the DFT calculations (Figure 6d). As shown in Figure 6b, the HOMO of CNM is primarily localized around nitrogen atoms, while the LUMO (Figure 6c) is centered on carbon atoms, indicating spatial separation that may limit charge mobility. Upon Cl doping, the HOMO becomes localized primarily around Cl and adjacent N atoms, while the LUMO is redistributed near the same region, mainly involving N and C atoms adjacent to Cl (Figure 6e,f). This spatial reorganization of frontier orbitals indicates enhanced orbital overlap and electronic polarization, which promotes more efficient charge carrier separation and transfer. The spatial proximity of HOMO and LUMO sites reduces the diffusion length required for charge migration, thereby minimizing recombination losses. These electronic modifications contribute directly to the improved photocatalytic activity observed for CNM–Cl(0.4). These modifications collectively contribute to the observed improvement in photocatalytic degradation of RB5. The molecular ESP maps and DOS plots for the CNM and CNM-Cl are shown in Figure 6g,h, respectively. In the ESP maps, we observed that the pristine CNM (Figure 6g) exhibited a relatively uniform potential distribution with slightly negative regions around the N atoms (blue regions), indicating areas of higher electron density. The potential was mostly neutral across the structure, except for a minor localized negative charge at the center. In contrast, for CNM-Cl (Figure 6h), the ESP map displays a strong red region around the Cl atom, indicating significant accumulation of electron density and highlighting the electron-withdrawing character of chlorine. This intense localization of the negative electrostatic potential around Cl suggests that it alters the electronic environment of the CNM, making the nearby atoms more positively charged owing to a relative deficit of electrons.

**Figure 6:**
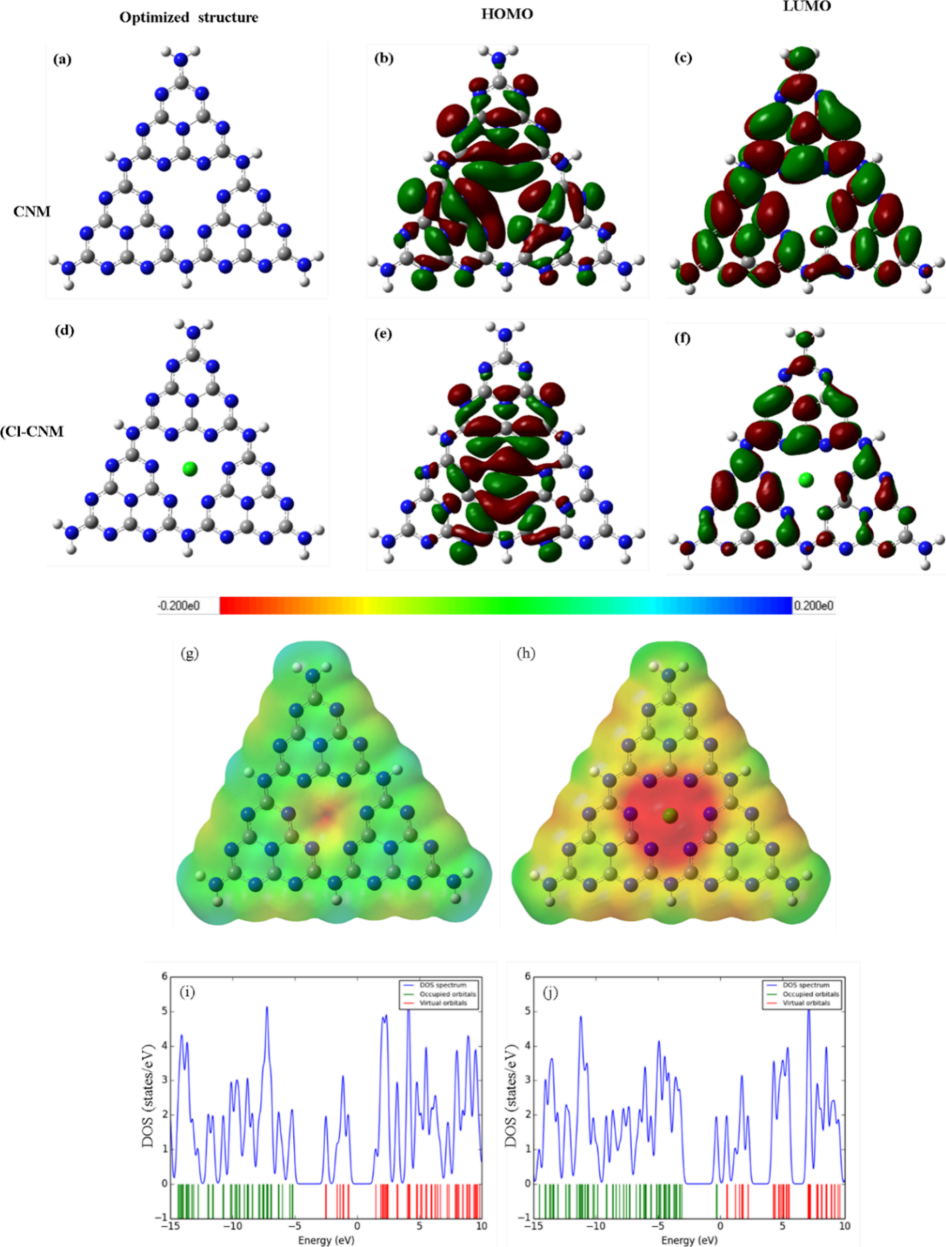
DFT-computed results for CNM and CNM-Cl: (a,d) optimized structures, (b,e) HOMO, and (c,f) LUMO, respectively. Molecular ESP maps and DOS plots for (g,i) CNM and (h,j) CNM-Cl, respectively. The blue, gray, light gray, and green colors represent N, C, H, and Cl atoms.

The DOS plots shown in Figure 6i,j provide further insights into the electronic properties of these materials. For the CNM (Figure 6i), the DOS spectrum shows a broad distribution of both occupied and unoccupied states. Interestingly, the separation between the occupied and unoccupied/virtual orbitals (2.63 eV) was closer to the experimental bandgap (2.87 eV), leading to the accurate and reliable use of the B3LYP/6-31G basis set. For CNM-Cl (Figure 6j), we observed a similar DOS pattern, but the inclusion of Cl introduced a slight shift in the energy levels. Specifically, the occupied orbitals (green spectrum) moved toward the virtual (red spectrum) with more pronounced peaks in the virtual orbitals above the Fermi level. This indicated that the incorporation of Cl altered the electronic states near the Fermi level, which likely influenced the conductivity and reactivity of the material. The shift and introduction of additional states in CNM-Cl suggests an altered electronic structure, which could affect the performance of the material in applications such as photocatalysis. Overall, the ESP and DOS analyses, along with the HOMO–LUMO, highlight how the inclusion of Cl in CNM significantly modifies both the electrostatic environment and the electronic structure of the material.

Furthermore, these computational findings support the experimental evidence suggesting that chlorine atoms are incorporated interstitially rather than through covalent bonding to the carbon or nitrogen atoms of the g-C_3_N_4_ framework. The lack of chemical shifts in XRD and FTIR spectra, along with the Cl 2p binding energy observed in XPS (∼198.94 eV), point toward nonbonded Cl^–^ ions. The electron density distributions in DFT simulations similarly reveal localized electronic effects without any indication of bond formation. This interstitial doping behavior is also consistent with prior studies on Cl-doped g-C_3_N_4_ systems,^63^ further validating our proposed mechanism.

### 3.5 Effects of Initial pH

The pH of the solution significantly influences the surface charge of both the catalyst and dye molecules, thereby impacting the number of active sites available on the catalyst.^77,78^ Figure S4 illustrates the impact of solution pH on photodegradation efficiency. The data showed that dye removal was more effective under acidic conditions (pH 4) than under neutral or alkaline conditions. This enhanced efficiency in acidic environment can be explained by the increased positive charge on the catalyst’s surface due to the adsorption of h^+^ ions as the pH decreases. This positive charge enhances the adsorption of negatively charged dye molecules, leading to a higher rate of RB5 degradation.

Figure S5 illustrates the pseudo-first-order kinetic graphs of RB5 degradation at various pH levels, with linear fits provided for each pH. The plot shows that the degradation rate was the highest at pH 4, as indicated by the steepest slope, and then decreased as the pH increased. Figure S6 shows a bar graph of the rate constants estimated from these plots, demonstrating that the rate constant at pH 4 was much larger than that at other pH values. Table S3 provides a summary of the initial and final pH values and the rate constants used in the degradation process. The table demonstrates that the catalytic efficiency was optimal under more acidic conditions, with a pH of 4 yielding the highest rate constant (0.107 min^–^^1^). These findings suggest that the solution pH has a significant influence on the surface interactions between the catalyst and dye molecules, thus affecting the overall degradation efficiency. Recent studies support these results, underscoring the importance of optimizing pH conditions to enhance photocatalytic efficiency.^79^

### 3.6 Determining Reactive Oxygen Species

Reactive oxygen species (ROS), was used to investigate the substances involved in the reaction, including ROS. The results are presented in Figure S7 revealed that the •OH–DMPO adduct exhibited quartet peaks with an intensity ratio of 1:2:2:1 across various chloride-doped C_3_N_4_ catalysts.^80,81^ Although photocatalytic g-CN materials are typically associated with the generation of superoxide radicals (•O_2_^–^), our experimental findings suggest that ROS primarily consist of hydroxyl radicals (•OH). In addition, the data in Figure S7 suggest that the •OH signal, characterized by a lower peak intensity, may originate from the decomposition of H_2_O_2_, which is produced from the reduction of water by •O_2_.^82^

### 3.7 Mechanism of Photocatalytic Activities

The photodegradation mechanism typically involves the generation of ROS, such as hydroxyl radicals (•OH), holes (h^+^), and superoxide radicals (O_2_^•–^), which play a crucial role in the degradation of organic dyes.^83–85^ Scheme 1 presents a model for the degradation of organic pollutants in water using a photocatalyst. When light energy strikes the catalyst and matches its bandgap, it excites a photoelectron (*e* ^*–*^) into the CB, leaving behind a hole (*h* ^*+*^) in the VB, as shown in eq 1). The electron–hole pair then participates in redox reactions: the hole oxidizes water and hydroxide ions (H_2_O/OH^–^) to produce hydroxyl radicals (•OH), as depicted in eq 2), whereas the photoexcited electrons reduce oxygen (O_2_) to form superoxide radicals (•O_2_^–^), as described in eq 3). Further electron capture by O_2_^–^ results in the formation of hydrogen superoxide (HOO^–^), as shown in eq 4). These highly reactive species (OH, •O_2_^–^, and HOO^–^) efficiently degrade and mineralize organic pollutants into smaller, nontoxic molecules like CO_2_ and H_2_O, as illustrated in Eqns.^5^ and,^6^ respectively:

$$\mathrm{Catalyst}+\mathrm{h\upsilon}\to e^{-}+h^{+}$$ 1

$${\mathrm{OH}}^{-}+h^{+}\to •\mathrm{OH}$$ 2

$$O_{2}+e^{-}\to •{O_{2}}^{-}$$ 3

$$•{O_{2}}^{-}+H_{2}O\to \mathrm{HOO}+{\mathrm{OH}}^{-}$$ 4

$$\mathrm{RB}5+•\mathrm{OH}\to {\mathrm{CO}}_{2}+H_{2}O$$ 5

$$\mathrm{RB}5+{O_{2}}^{-}/\mathrm{HOO}\to {\mathrm{CO}}_{2}+H_{2}O$$ 6

### 3.8 Total Organic Compound Analysis

Total Organic Compound (TOC) analysis (Figure S8) provide a comprehensive evaluation of the photocatalytic performance. While UV–vis analysis effectively demonstrated the decolorization of RB5, TOC analysis revealed critical insights into the extent of mineralization during the photocatalytic process. The negligible TOC removal in the absence of a catalyst (0.00%) confirms that photolysis alone is insufficient for degradation. The CNM catalyst achieved 18.42% TOC removal, whereas CNM–Cl(0.4) reached 24.98%, indicating that chlorine doping significantly enhances the photocatalytic breakdown of RB5 into smaller, less carbon-rich intermediates or CO_2_. These findings confirm that the observed color loss was not merely due to molecular fragmentation but was accompanied by partial oxidation and mineralization of the organic content. Therefore, the integration of UV–vis and TOC analyses offers a more reliable assessment of photocatalytic degradation pathways and underscores the superior mineralization capability of CNM–Cl(0.4) in wastewater treatment applications.

### 3.9 Photocatalytic Stability and Structural Integrity

The structural and compositional stability of CNM and CNM-Cl(0.4) after photocatalytic reuse was evaluated using SEM and XRD analyses. To evaluate the structural stability of the catalysts, SEM images were collected for CNM and CNM-Cl(0.4) before and after two photocatalytic cycles (Figure S9a–d). There is no significant variation in the postreaction morphologies compared to the pristine samples, indicating the structural robustness of the materials during reuse. To address this important aspect further, XRD patterns of both CNM and CNM-Cl(0.4) before and after two photocatalytic cycles are provided in Figure S10. As shown in Figure S10, the XRD patterns of both catalysts exhibit no noticeable shift in peak positions or variation in intensities, indicating that the crystalline structure is well preserved following the recycling process. These results further support the structural and compositional stability of the catalysts under the applied reaction conditions.

## 4 Conclusions

In this study, the impact of Cl doping on CNM was thoroughly investigated with respect to the photocatalytic effectiveness of RB5 pollutant degradation. From the XRD analysis, the presence of two distinct peaks indicated the successful synthesis of the CNM catalyst. The doping of Cl atoms into the CNM reduced the particle size of the as-prepared CNM, which may enhance the active sites of the catalyst. Interestingly, from the XRD analysis, we observed that interstitial Cl doping provided synergetic effects and did not deteriorate the original CNM structure. We conducted a quantitative assessment of the photocatalytic performance of undoped and Cl-doped CNM using a pseudo-first-order model to calculate the rate constant. Notably, the addition of 0.4 g of ammonium chloride to the CNM consistently demonstrated superior photocatalytic activity in degrading RB5. The generated photocurrent suggests that Cl doping in carbon nitride significantly enhances the current production, promotes charge separation, and reduces the rate of recombination. Using sunlight and advanced materials, photodegradation can significantly mitigate the impact of organic pollutants on water resources, thereby supporting sustainable clean water. DFT calculations of the electronic properties revealed that the inclusion of Cl atoms in the CNM significantly affected the ESP and DOS results. This study presents a promising method to enhance the efficacy of CNM–Cl in addressing environmental pollution.

Importantly, the synthesis approach for the Cl-doped CNM (CNM–Cl(0.4)) is inherently scalable and environmentally friendly. It involves a simple, low-cost, solid-state thermal treatment using readily available precursors (melamine and ammonium chloride). This method is conducive to potential large-scale production without the need for complex solvents or hazardous reagents. Moreover, the catalyst exhibits excellent structural stability and reusability across multiple cycles, as confirmed by SEM and XRD results (Figures S9 and S10), further supporting its suitability for continuous industrial operation. This work suggests that future studies may consider integrating the CNM–Cl(0.4) catalyst into immobilized or flow-type photocatalytic reactors for practical water purification under solar light.

## Supplementary Material



## Notes

Appearance of Cl-doped into CNM synthesized photocatalyst with (a) 0.1, (b) 0.2, (c) 0.3, (d) 0.4, and (e) 0.5 g of Cl; calibration curve for RB5 using UV–vis spectroscopy; recycling test of RB5 photodegradation using CNM and CNM-Cl(0.4) catalysts. (RB5: 50 ppm; catalyst: 250 mg/L; light source: 420 nm LED and 11,300 Lux); effects pH on the photodegradation of RB5 using of CNM-Cl (0.4) catalyst. (catalyst: 250 mg/L, RB5:50 ppm, LED 420 nm 1130 Lux); pseudo first order kinetics versus pH of CNM-Cl(0.4) catalyst on the degradation of the RB5; rate constant versus pH of CNM-Cl(0.4) catalyst aqueous solution; electron pa aramagnetic resonance (EPR) spectra of CNM-Cl ([DMPO]: 25 mM, irradiation time: 20 min); TOC removal efficiency of RB5 after 40 min of LED irradiation using CNM and CNM–Cl(0.4) photocatalysts; SEM images of (a,c) CNM and (b,d) CNM-Cl(0.4), respectively, before and after two round recycle test; XRD patterns of CNM and CNM-Cl(0.4) before and after two recycle test; band gap, rate constant, BET surface area of the CNM and CNM-Cl(0.4) catalysts; and comparative summary of RB5 photocatalytic degradation using various catalysts under different experimental conditions; and catalyst solution pH before and after photodegradation of RB5 by CNM-Cl(0.4) and the reaction rate constants (PDF)
