# Supplementary material for: Chlorine-Doped Graphitic Carbon Nitride for Enhanced Photocatalytic Degradation of Reactive Black 5: Mechanistic and DFT Insights into Water Remediation
Source: ACS Omega. 2025 Aug 27;10(35):39861–74. doi: 10.1021/acsomega.5c04017 (PMC12423792; doi:10.1021/acsomega.5c04017)
Supplement: Supplementary file 1 [file ao5c04017_si_001.pdf]

Supporting Information

Chlorine-doped graphitic carbon nitride for enhanced photocatalytic degradation of reactive Black 5: Mechanistic and DFT insights toward water remediation

Jau-Min Ji†, Tesfaye Abebe Geleta†, Yang-hsin Shih*, and Tee Ren Qian

Department of Agricultural Chemistry, National Taiwan University, No. 1, Sec. 4, Roosevelt Road, Taipei 106, Taiwan

* Corresponding author: Yang-hsin Shih (yhs@ntu.edu.tw)

† These authors contributed equally

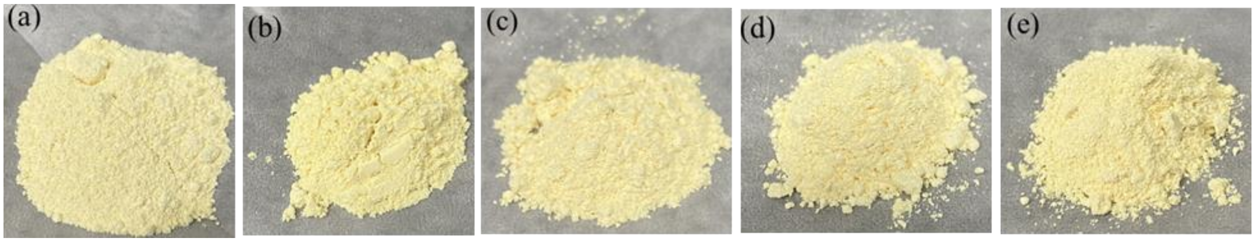

Fig. S1. The appearance of Cl-doped into CNM synthesized photocatalyst with (a) 0.1, (b) 0.2, (c) 0.3, (d) 0.4, and (e) 0.5 g of Cl.

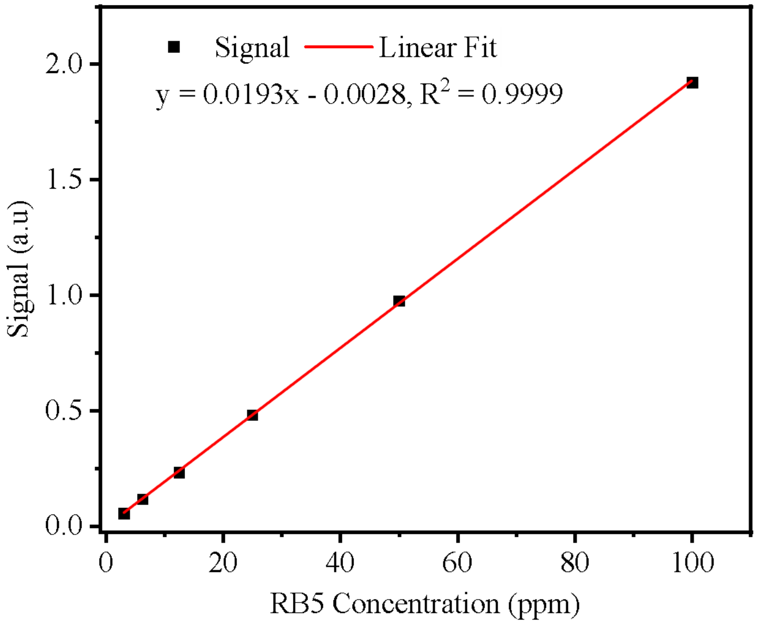

Fig. S2. The calibration curve for RB5 using UV-vis spectroscopy.

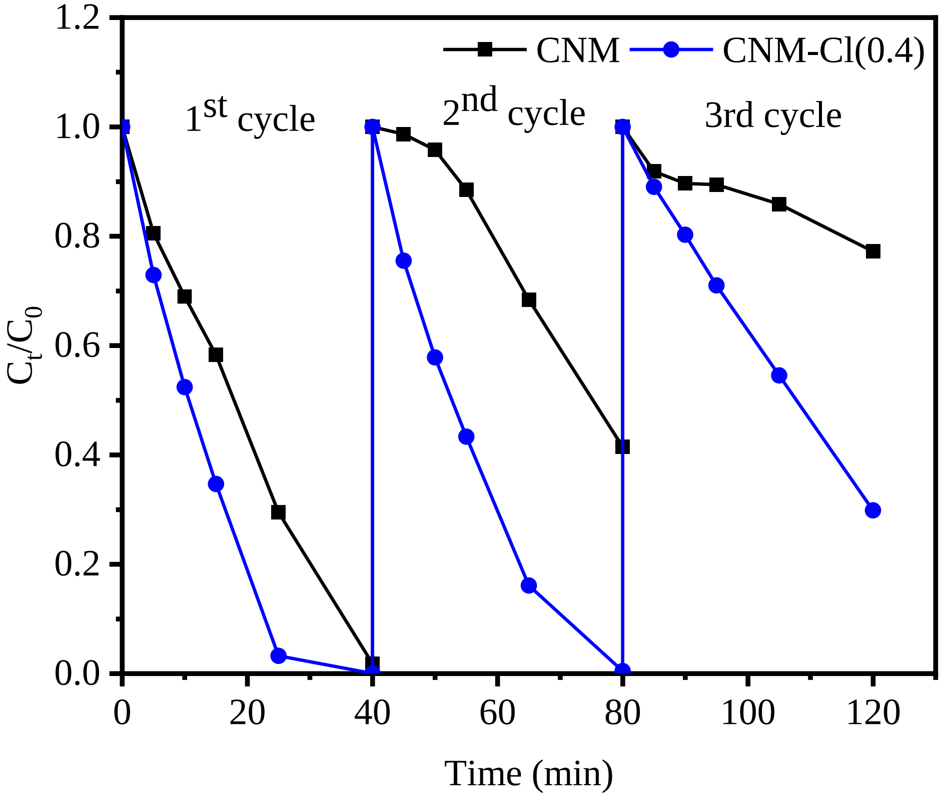

Fig. S3. Recycling test of RB5 photodegradation using CNM and CNM-Cl(0.4) catalysts. (RB5: 50 ppm; catalyst: 250 mg/L; light source: 420 nm LED and 11,300 Lux).

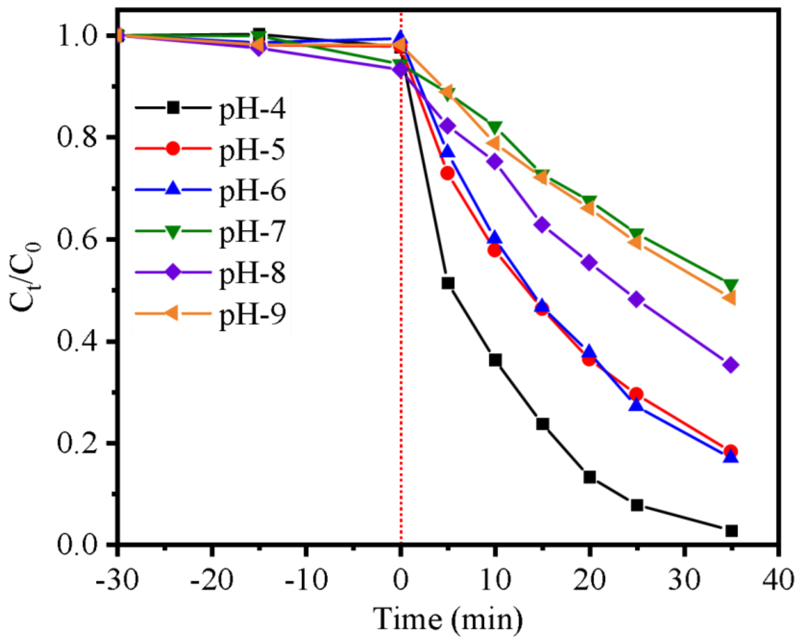

Fig. S4. The effects pH on the photodegradation of RB5 using of CNM-Cl (0.4) catalyst. (catalyst: 250 mg/L, RB5: 50 ppm, LED 420 nm 1130 Lux).

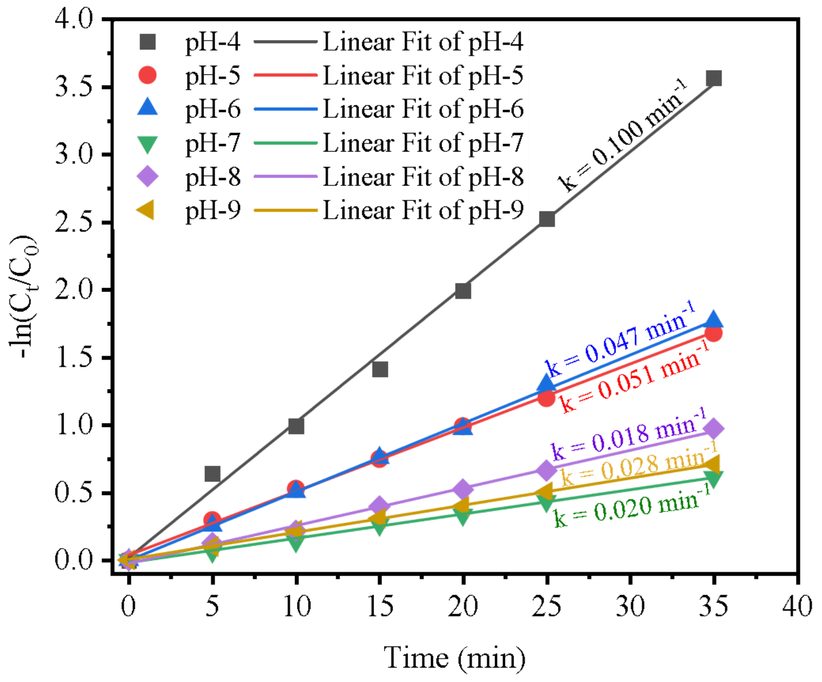

Fig. S5. Pseudo first order kinetics versus pH of CNM-Cl(0.4) catalyst on the degradation of the RB5.

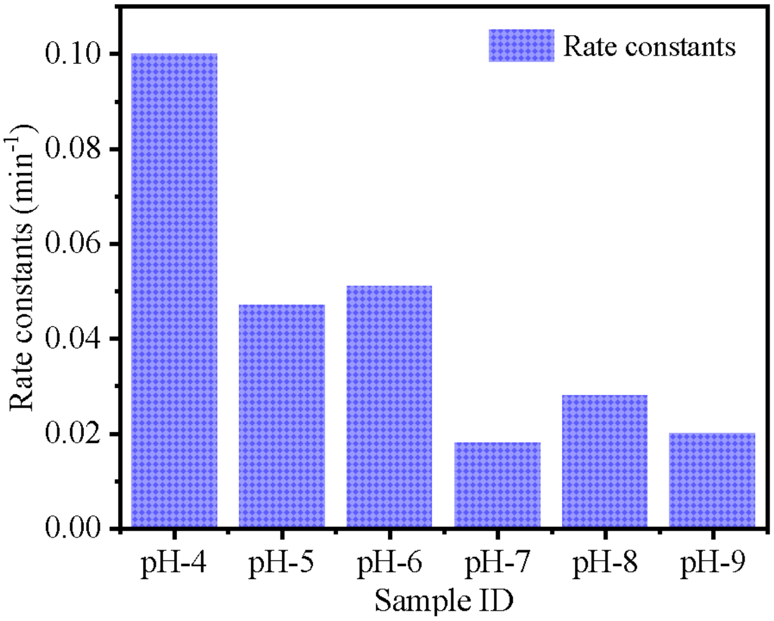

Fig. S6. The rate constant versus pH of CNM-Cl(0.4) catalyst aqueous solution.

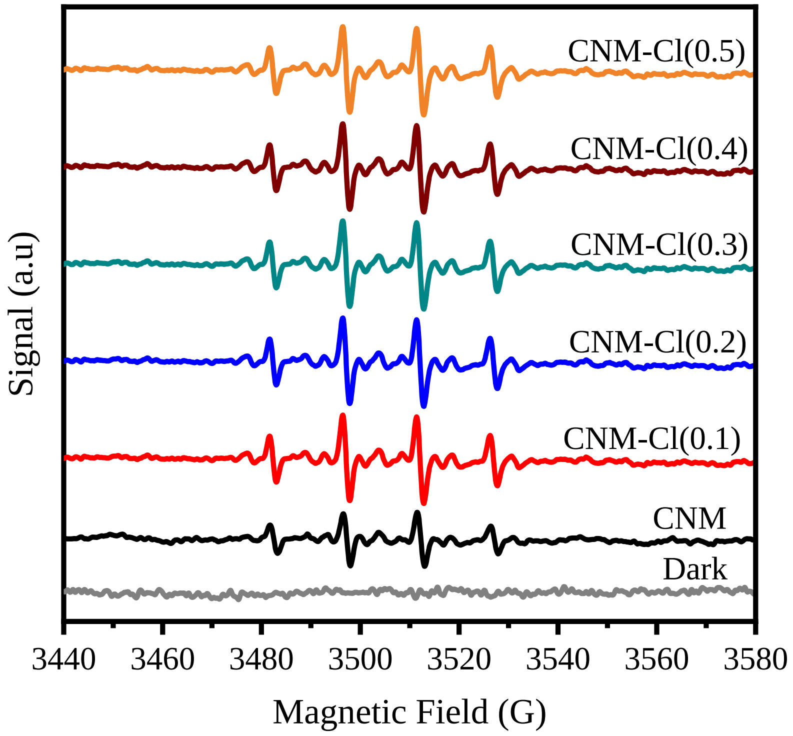

Fig. S7. Electron-paramagnetic resonance (EPR) spectra of CNM−Cl ([DMPO]: 25 mM, irradiation time: 20 min).

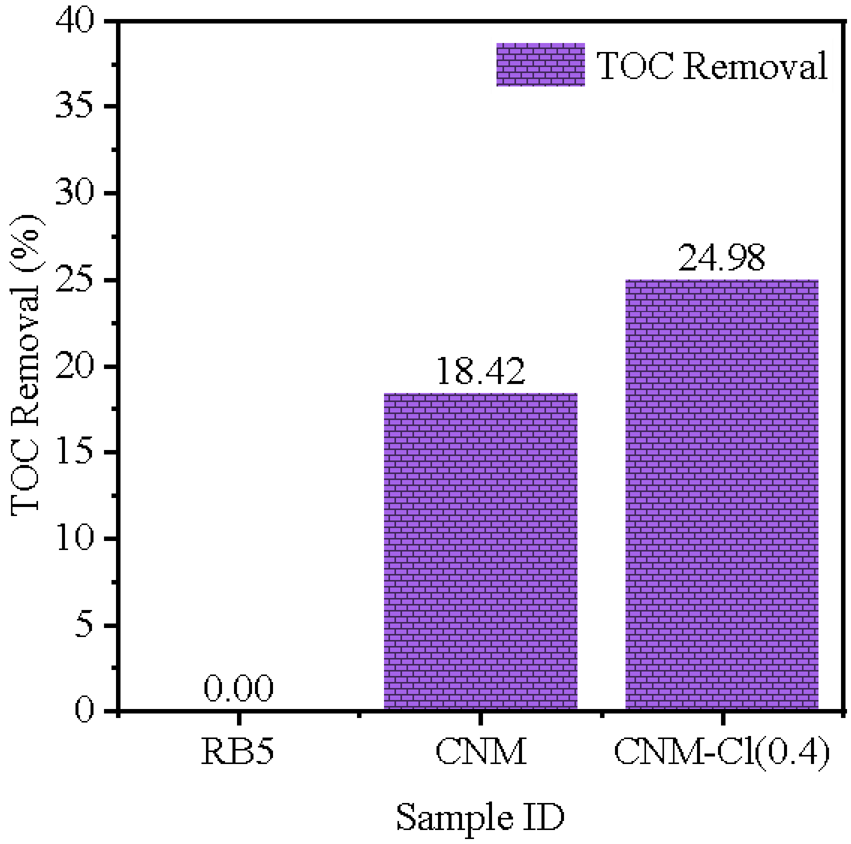

Figure S8. TOC removal efficiency of RB5 after 40 minutes of LED irradiation using CNM and CNM–Cl(0.4) photocatalysts.

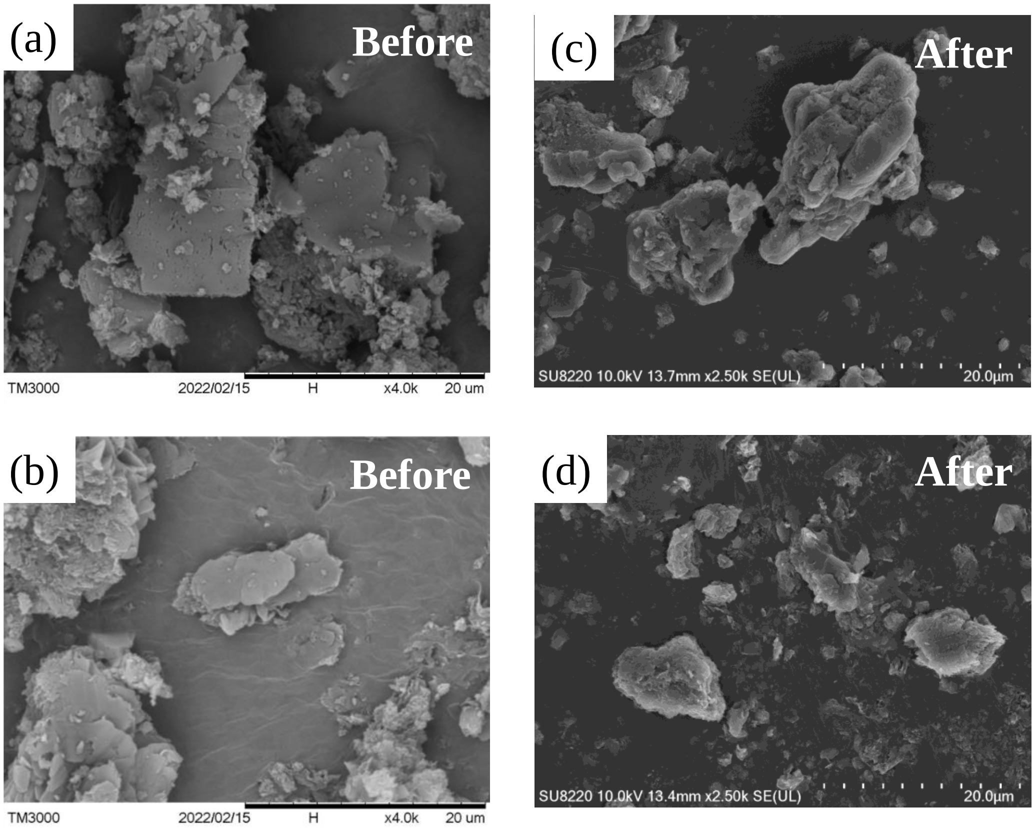

Figure S9. SEM images of (a,c) CNM and (b,d) CNM-Cl(0.4), respectively, before and after two round recycle test.

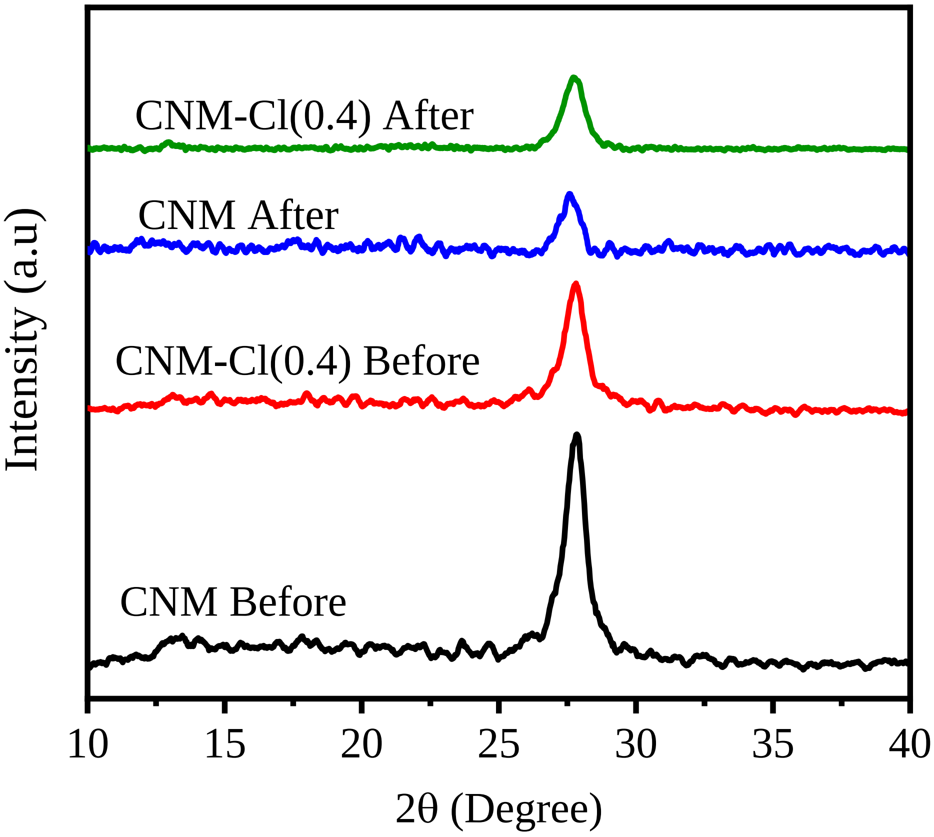

Figure S10. XRD patterns of CNM and CNM-Cl(0.4) before and after two recycle test.

Table S1. Band gap, rate constant, and BET surface area of the CNM and CNM-Cl(0.4) catalysts.

Catalyst	Band gap (eV)	Rate constant (min^{-1})	BET specific surface area (m2/g)
CNM	2.87	0.113	11.22
CNM-Cl(0.4)	2.83	0.199	44.68

Table S2. Comparative summary of RB5 photocatalytic degradation using various catalysts under different experimental conditions.

Catalyst	Light source	Catalyst concentration (mg/L)	RB5 concentration (ppm)	Reaction time (min)	Rate constant (min^{-1})	Degradation efficiency (%)	Ref.
rGO	96 W UV-A lamp	300	10	60	0.01395	56	[1]
TiO_2-coated PET	15 W LED lamp	500	20	120	0.0328	99.99	[2]
WO_3/TiO_2	150 W LED	250	30	120	0.0439	92	[3]
Ag/ZnO	100 W LED	500	10	780	0.0017	74	[4]
CNM-Cl(0.4)	150 W LED	250	50	25	0.199	99	This work

Table S3. Catalyst solution pH before and after photodegradation of RB5 by CNM-Cl(0.4) and the reaction rate constants.

Adjusted pH	Initial pH	Final pH	Rate constant (min^{-1})
4	4.8	4	0.100
5	5.7	5.8	0.047
6	6.6	5.7	0.051
7	6.9	6.1	0.018
8	7.6	6.2	0.028
9	6.9	6.3	0.020

Reference

1. Wong, C.P.P.; Lai, C.W.; Lee, K.M.; Abd Hamid, S.B. Advanced chemical reduction of reduced graphene oxide and its photocatalytic activity in degrading reactive black 5. Materials, **2015**. *8*(10), 7118-7128.

2. Mohammadi-Galangash, M.; Mousavi, S.-K.; Shirzad-Siboni, M. Photocatalytic degradation of reactive black 5 from synthetic and real wastewater under visible light with TiO2 coated PET photocatalysts. Scientific Reports, **2025**. *15*(1), 14314.

3. Chau, J.H.F.; Lee, K.M.; Pang, Y.L.; Abdullah, B.; Juan, J.C.; Leo, B.F.; Lai, C.W. Photodegradation assessment of RB5 dye by utilizing WO3/TiO2 nanocomposite: a cytotoxicity study. Environmental Science and Pollution Research, **2022**. *29*(15), 22372-22390.

4. Santos, P.B.; Santos, J.J.; Corrêa, C.C.; Corio, P.; Andrade, G.F.S. Plasmonic photodegradation of textile dye Reactive Black 5 under visible light: a vibrational and electronic study. Journal of Photochemistry and Photobiology A: Chemistry, **2019**. *371*, 159-165.
